# Are National Breast and Cervical Cancer Early Detection Program Recipients Providing Services in Counties Heavily Burdened by Breast and Cervical Cancer?

**DOI:** 10.3390/ijerph21020188

**Published:** 2024-02-07

**Authors:** Yamisha Bermudez, Lia Scott, Jacqueline Miller, Amy DeGroff, Michele Beckman

**Affiliations:** 1 Totally Joined for Achieving Collaborative Techniques, Atlanta, GA 30303, USA; 2 Department of Population Health Sciences, School of Public Health, Georgia State University, Atlanta, GA 30303, USA; 3 Program Services Branch, Division of Cancer Prevention and Control, National Center for Chronic Disease Prevention and Health Promotion, Centers for Disease Control and Prevention, Atlanta, GA 30341, USA

**Keywords:** cancer burden, spatial clustering, cancer screening program

## Abstract

Introduction: Alignment of National Breast and Cervical Cancer Early Detection Program (NBCCEDP) clinical services with the spatial distribution of breast and cervical cancer burden is essential to maximizing programmatic impact and addressing cancer disparities. This study identified spatial clustering of breast and cervical cancer burden scores and assessed whether and to what extent NBCCEDP clinical services were associated with clusters for the 5-year period, 2015–2019. Methods: We examined burden scores for spatial clustering using Local Indicators of Spatial Association (LISA) tests in GeoDA. We then used *t*-tests to compare the NBCCEDP 5-year average percentage of eligible women served clinical breast and cervical cancer services between hotspot (high burden) and coolspot clusters. Results: There was statistically significant spatial clustering in the pattern of breast and cervical cancer burden scores across counties, with hotspot clusters mostly observed in the Southern region, Idaho and Nevada. For both breast and cervical cancer, higher percentages of eligible women received breast and cervical cancer clinical services in coolspot clusters compared to hotspot clusters during each year from 2015–2019. Conclusion: NBCCEDP clinical services can help reduce breast and cervical cancer burden. Yet, during 2015–2019, increased service delivery was not aligned with the spatial distribution of counties with greater breast and cervical cancer burdens. NBCCEDP recipients may improve their impact on breast and cervical cancer burden by prioritizing and consistently increasing service delivery in cancer burden hotspot clusters if they have not already maximized their resources in these areas.

## 1. Introduction

Cancer is the second leading cause of mortality in the United States [1]. In 2019, 16% of cancer deaths among women were due to breast and cervical cancer combined [1]. Early detection through screening for breast and cervical cancer can significantly reduce cancer mortality [2,3]. Yet 50% of women with cervical cancer and more than 30% of women with breast cancer are diagnosed at a late stage (regional and distant) [4]. Socio-economic and insurance status are significant predictors of breast and cervical cancer screening. Women without health insurance and with lower incomes have significantly lower breast and cervical cancer screening rates than women with insurance and higher incomes [5].

In 1991, the National Breast and Cervical Cancer Early Detection Program (NBCCEDP) was established to address barriers to screening and improve cancer outcomes [6]. The NBCCEDP provides breast and cervical cancer screening, diagnostic testing, and referral for cancer treatment to women with income at or below 250% of the federal poverty level (FPL) and who are underinsured or uninsured. Since its establishment, NBCCEDP has served more than 6.2 million eligible women and provided more than 16.1 million breast and cervical cancer examinations. Recipients of the NBCCEDP, which are funded by the Centers for Disease Control and Prevention (CDC), currently include 50 states, the District of Columbia, 13 tribes or tribal organizations, 2 US territories, and 5 US-affiliated Pacific Islands.

One of NBCCEDP’s key strategies is to prioritize service delivery among populations disproportionately burdened by breast and cervical cancer. Cancer burden is often defined as cancer incidence or mortality [7,8,9,10]. However, researchers at the Kentucky Cancer Registry suggest using composite burden scores that consider cancer incidence, socio-demographic characteristics, and risk behaviors together. According to their logic model, socio-demographic characteristics (i.e., poverty, insurance) are known to influence cancer screening, which in turn affects cancer incidence and mortality [11]. Thus, using a summative cancer burden score that accounts for these factors together allows for more accurate identification of highly burdened locations. Similarly, spatial cluster analysis is also an efficient method for identifying highly burdened places. Specifically, spatial cluster analysis can identify counties with above average breast and cervical cancer burden scores that share boundaries with neighboring counties that also have above average burden scores (i.e., hotspot clusters) and counties with below average breast and cervical cancer burden scores that share boundaries with neighboring counties that also have below average burden scores (i.e., coolspot clusters). Hotspot clusters could be prioritized for public health intervention, guiding prevention strategies, and informing policy implementation [12]. Together, these methods may help to identify counties with the greatest breast and cervical cancer burden, which could be ideal places for prioritizing the distribution of NBCCEDP clinical services.

Using these methods, we identified spatial clustering of composite breast and cervical cancer burden scores and assessed whether and to what extent NBCCEDP clinical services were associated with clusters over the 5-year period, 2015–2019. We specifically aimed to:

1. Generate breast and cervical cancer burden scores for each US county.
2. Identify spatial clusters of counties with higher than average and lower than average breast and cervical cancer burden scores (i.e., hotspot clusters and coolspot clusters).
3. Determine whether clusters were associated with the 5-year average percentages of eligible women served through the NBCCEDP.
4. Examine the yearly change in the average percentage of eligible women served in hotspot and coolspot clusters from 2015–2019.

NBCCEDP recipients use cancer outcome data to identify populations in greatest need, specifically those with higher late stage cancer diagnoses and mortality. After NBCCEDP recipients have assessed the variation in breast and cervical cancer burden, ideally, this information is used to inform where in their state or jurisdiction to prioritize clinical services. Therefore, we would expect that the county-level distribution of those served by the NBCCEDP aligns with the spatial distribution of the burden of breast and cervical cancer across counties. Specifically, it was our hypothesis that counties with a higher breast and cervical cancer burden would also have a greater percentage of eligible women served by the NBCCEDP. An analysis exploring this relationship across all program-funded states has never been conducted.

Assessing NBCCEDP service delivery in identified hotspot clusters could provide useful information for NBCCEDP awardees to determine whether they have been targeting the best places for implementing clinical services or if they should consider alternative places to focus their efforts. Results may also inform the CDC’s future allocation of NBCCEDP resources. Ensuring that NBCCEDP screening services are available in high burden areas could improve programmatic impact and help to alleviate cancer disparities.

## 2. Materials and Methods

This cross-sectional study used NBCCEDP program data that were collected for every person receiving clinical services through the NBCCEDP and reported to CDC [13]. Each record describes the screening test received, diagnostic follow-up of abnormal test results if needed, treatment initiation if diagnosed with cancer, and other clinical outcomes. Records also describe patient demographic characteristics, including county or ZIP Code of residence (patients have the choice of which they would like to report). Using records for clinical services delivered during 2015–2019, we geocoded each record to their county of residence, using the reported county of residence or the ZIP Code using a ZIP-County crosswalk [14]. We then assessed the county-level 5-year average number of women served (received NBCCEDP breast and cervical cancer screening and/or diagnostic service), separately, for the period 2015–2019. Data from 46 of the 70 NBCCEDP recipients (46 states) were included in the analysis. We excluded tribes, tribal organizations, US territories, US-affiliated Pacific Islands, the District of Columbia, Alaska, Hawaii, Minnesota, and Kansas due to insufficient spatial data. We also excluded 94 breast (0.01%) and 99 (0.01%) cervical clinical records with inaccurately reported ZIP Codes.

We used 2015–2019 Small Area Health Insurance Estimates (SAHIE) data to determine the percentage of women eligible for the NBCCEDP program [15]. SAHIE data provide model-based state and county estimates of the number of individuals with and without health insurance [15]. At both the state and county levels, insurance coverage data are reported by age, sex, and income group based on FPL. Using county-level reports, we calculated the annual and 5-year average number of women meeting NBCCEDP eligibility requirements for 2015–2019 based on age, income, and insurance status. Women met the NBCCEDP eligibility requirement if they were uninsured, aged 40–64 years for breast screening, aged 21–64 years for cervical screening, and lived in households with incomes at or below the state-established FPL. Thirty-seven state programs set income eligibility criteria at 250% FPL, 12 at 200% FPL, 1 at 225% FPL, and 1 at 185% FPL. We determined the county-level percentage of NBCCEDP-eligible women served by dividing the county-level number of women who received clinical services through the NBCCEDP by the county-level number of women eligible for the NBCCEDP. This was calculated separately for breast and cervical cancer for each year, 2015–2019. Next, we calculated the county-level, 5-year average percentage of eligible women served for both breast and cervical cancer.

Additionally, we obtained data describing county-level, age-adjusted percentages of women of all races (aged 50–74 for breast and 21–65 for cervical) not up-to-date with mammograms and Pap tests for the years 2016 and 2018 from the Behavioral Risk Factors Surveillance System (BRFSS) [16,17]. We also obtained county-level late stage incidence data describing the age-adjusted rate of regional and distant breast and cervical cancer diagnoses among women of all races (aged 40–64 for breast and 20–64 for cervical) for the years 2015–2019 from the United States Cancer Statistics (USCS) [18]. Finally, we obtained data describing county-level percentage of women of all races (aged 35–64 for breast and 18–64 for cervical) with income below FPL in the past 12 months for the years 2015–2019 from the American Community Survey (ACS) [19]. 

We generated separate breast and cervical cancer burden scores for each US county by summing the standardized and weighted averages of late stage cancer incidence, not up-to-date with screening, and poverty measures for the 5-year period, 2015–2019. This approach adopts the logic model behind Kentucky Cancer Registry researchers composite scores; however, we used a modified approach to calculate our scores. Researchers at the Kentucky Cancer Registry observed values for each selected factor and ranked them separately for each Area Development District (ADD), then the ranks for the equally weighted factors in each ADD were summed to create the score. Since the level of study is counties rather than a small number of ADDs, we did not find it appropriate to rank each factor across more than 3000 counties. Instead, we weighted and standardized factors. First, we calculated county-level 5-year averages for late stage cancer incidence and poverty variables separately using SAS version 9.4 [20]. The age-adjusted BRFSS cancer screening measures are only available for 2 years of the 5-year study period (2016 and 2018), so for these measures we calculated a county-level 2-year average. The county-level 5-year average factors and 2-year average factors were then standardized using Z-scores to normalize each of the variables. Next, we used the Direct Assignment Technique (DAT) to assign weights for each of the three factors. We divided a fixed number of points (100 points) among each of the factors based on their absolute importance relative to the standard reference point (cancer mortality). Relative to mortality, we decided that the order of importance from greatest to least was late stage cancer incidence (45 points), percent not up-to-date with screening (35 points), and percent poverty (20 points). Weights were obtained by dividing each individual factor’s points by the sum of points across all attributes. We then multiplied the standardized value of each county-level factor by its respective weight. For each county in the United States, the 3 standardized and weighted factors were summed to generate county-level breast and cervical cancer burden scores separately. Appendix A demonstrates the burden score calculation for a single county. 

Next, we used GeoDa version 1.6.0 to assess the global and local spatial clustering of county-level breast and cervical cancer burden scores [21]. The Moran’s I test first determined if there was global clustering, and based on a significant Moran’s I test statistic, we rejected the hypothesis of spatial randomness, which predicated the use of the Local Indicators of Spatial Association (LISA) test for the identification of local clusters [22,23]. The LISA test first calculates a test statistic for each county, representing whether the county has a statistically significantly higher or lower burden score compared to the national average burden score [23]. This can result in 4 types of spatial clusters: high-high (counties with higher than average measures adjacent to counties with higher than average measures known as hotspot clusters), low-low (counties with lower than average measures adjacent to counties with lower than average measures known as coolspot clusters), high-low, and low-high. Spatial adjacency among locations for the LISA test was defined using a spatial weights matrix. The spatial weights matrix was created using queen contiguity weights, which define a location’s neighbors as those with either a shared border or vertex/corner. To determine the statistical significance of LISA test statistics, GeoDA uses bootstrapping, a permutation approach. Using statistically significant LISA test statistics, we created 2 separate LISA cluster maps in QGIS version 3.16.9 for breast and cervical cancer [24]. These maps show all 4 cluster types. However, we focused on 2 cluster types for comparison in the remainder of our analyses: counties in high-high clusters (hereafter called hotspot clusters) and low-low clusters (hereafter called coolspot clusters).

Using SAS, we employed 2 independent sample *t*-tests, for breast and cervical, to test for statistically significant mean differences in the 5-year average percentage of eligible women served between counties in hotspot and coolspot clusters, at significance level 0.05 [20]. Lastly, we assessed the yearly percentage of eligible women served for breast and cervical cancer clinical services by the 2 cluster categories, hotspot and coolspot clusters, and displayed results in a line graph. Although 2020 cancer incidence data is available, this analysis focused on years 2015–2019 because it represents the most recent 5 years of available data prior to the impact of the COVID-19 pandemic.

## 3. Results

### 3.1. Breast Cancer

During 2015–2019, the US 5-year average, age-adjusted rate of late stage breast cancer cases among women of all races aged 40–64 was 72.15 per 100,000 women. During the same 5-year period, a US average of 2.95% of women of all races aged 35–64 reported income below FPL in the past 12 months. The US 2-year average (2016 and 2018) age-adjusted percentage of women aged 50–74 not up-to-date with breast cancer screening was 29.24%. The US 5-year average county-level breast cancer burden scores ranged from −1.6 to +7.3, with higher scores representing a greater burden.

**Figure 1:**
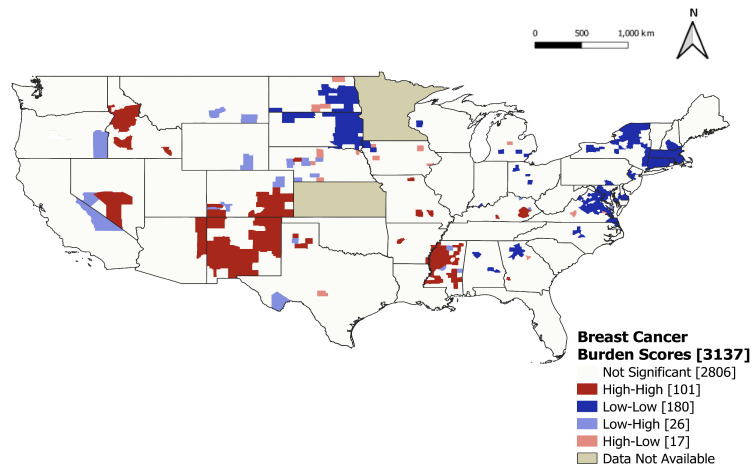
Results of a LISA cluster analysis on 5-year average breast cancer burden scores in US counties, 2015–2019. County-level breast cancer burden scores are divided into four categories: high-high (counties with higher than average measures adjacent to counties with higher than average measures), low-low (counties with lower than average measures adjacent to counties with lower than average measures), high-low, and low-high.

We concluded that there was global clustering in the patterns of the 5-year average breast cancer burden scores across counties (Moran’s I = 0.29; *p* < 0.01). LISA cluster results further identified 101 statistically significant hotspot clusters (colored red) and 180 statistically significant coolspot clusters (colored dark blue) (Figure 1). Hotspot clusters were observed in 13 states, with more in Colorado, Idaho, Mississippi, Nevada, and New Mexico. Coolspot clusters were observed in 17 states and were most apparent throughout the Midwest and Northeast regions of the United States.

**Figure 2:**
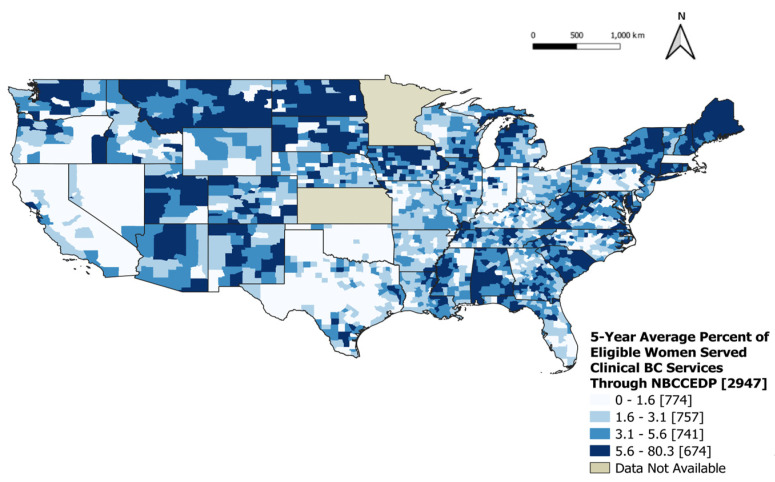
Five-year average percentage of eligible women served clinical breast cancer services through the NBCCEDP, 2015–2019. The 5-year average percentage of eligible women served clinical breast cancer services through NBCCEDP was divided into four categories: 0–1.6%, 1.6–3.1%, 3.1–5.6%, and 5.6–80%.

During 2015–2019, there were 14,483,700 women eligible to receive breast cancer clinical services through the NBCCEDP, and of those eligible, 1,164,138 were served through the program (8.03%). When looking at the geographic distribution of the 5-year average percentage of eligible women served for breast cancer clinical services through the NBCCEDP, greater percentages were evident in several counties throughout the states, including Connecticut, Iowa, Maine, Montana, Minnesota, North Dakota, New York, and Utah (Figure 2).

**Figure 3:**
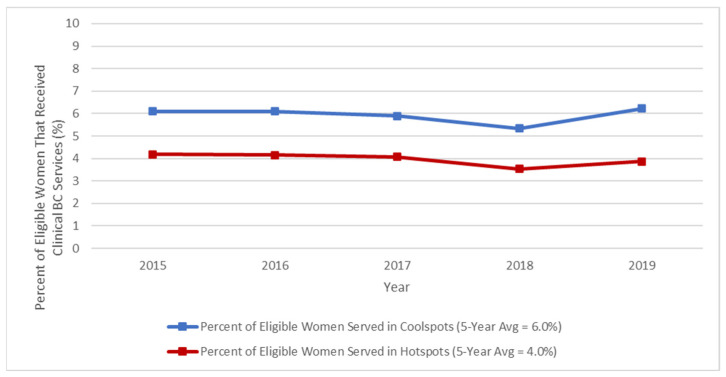
Yearly percentage of eligible women receiving clinical breast cancer services through the NBCCEDP in breast cancer hotspot vs. coolspot clusters, 2015–2019.

*T*-test results indicate there was a statistically significantly lower 5-year average percentage of eligible women served in breast cancer hotspot clusters compared to breast cancer coolspot clusters, 3.96% and 5.93%, respectively (tobs = 2.49, df = 279, *p* ≤ 0.05). Lastly, we found that among the observed 180 breast cancer coolspot clusters, the percentage of eligible women who received clinical NBCCEDP breast cancer services decreased from 6.1% in 2015 to 5.3% in 2018, with a slight increase to 6.2% in 2019. Among the 101 breast cancer hotspot clusters, the percentage of eligible women who received clinical breast cancer services remained steady at 4.1% from 2015–2017. However, in 2018, the percentage of eligible women who received clinical breast cancer services in hotspot clusters decreased slightly to 3.5%. By 2019, that percentage had increased back to 4%. Overall, the annual percentages of eligible women who received clinical breast cancer services were higher in coolspot clusters compared to hotspot clusters during each year from 2015–2019 (Figure 3).

### 3.2. Cervical Cancer

During 2015–2019, the US 5-year average age-adjusted rate of late stage cervical cancer cases among women of all races aged 20–64 was 5.6 per 100,000 women. During the same 5-year period, a US average of 5.42% of women of all races aged 18–64 reported income below FPL in the past 12 months. The US 2-year average (2016 and 2018) age-adjusted percentage of women aged 21–65 not up-to-date with cervical cancer screening was 16.12%. The US 5-year average county-level cervical cancer burden scores ranged from −1.7 to +6.3 with higher scores representing a greater burden. 

**Figure 4:**
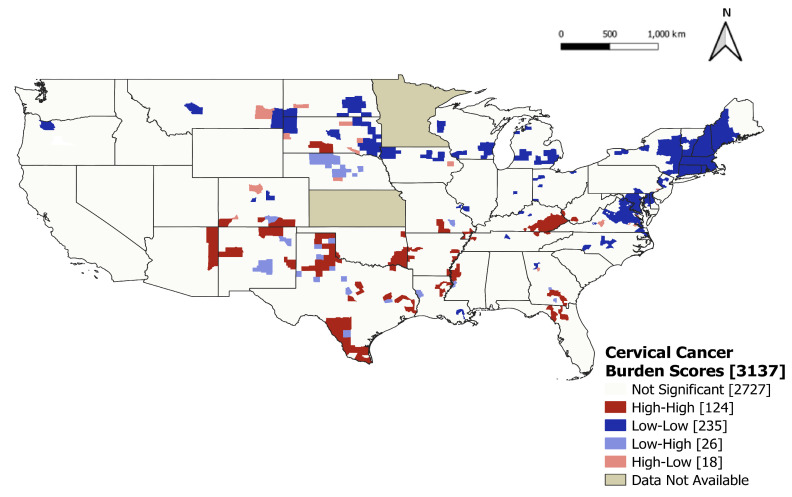
Results of a LISA cluster analysis on 5-year average cervical cancer burden scores in US counties, 2015–2019. County-level cervical cancer burden scores are divided into four categories: high-high (counties with higher than average measures adjacent to counties with higher than average measures), low-low (counties with lower than average measures adjacent to counties with lower than average measures), high-low, and low-high.

We concluded that there was global clustering in the patterns of the 5-year average cervical cancer burden scores across counties (Moran’s I = 0.36; *p* < 0.01). We found 124 statistically significant hotspot clusters (colored red) and 235 statistically significant coolspot clusters (colored dark blue) (Figure 4). Hotspot clusters were observed in 16 states and were most apparent throughout the Southern region of the United States, as well as in Arizona and New Mexico. Coolspot clusters were observed in 23 states and were most apparent throughout the Midwest and Northeast regions of the United States.

**Figure 5:**
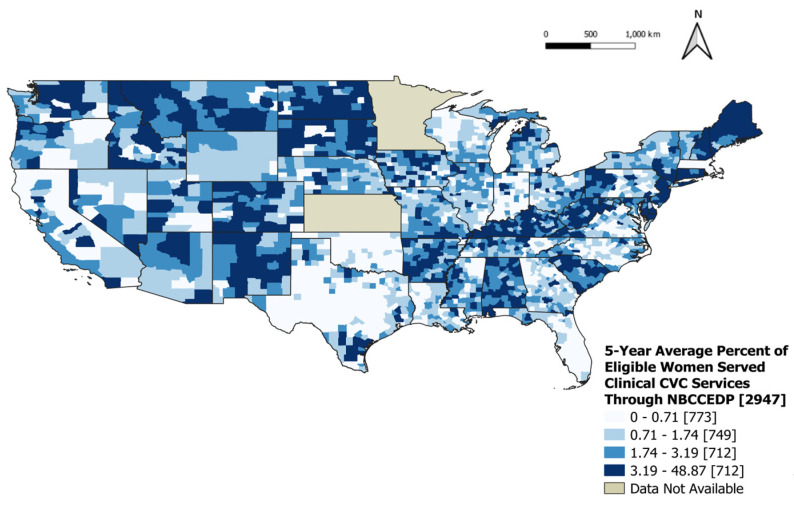
Five-year average percentage of eligible women served clinical cervical cancer services through the NBCCEDP, 2015–2019. The 5-year average percentage of eligible women served clinical cervical cancer services through NBCCEDP was divided into four categories: 0–0.71%, 0.71–1.74%, 1.74–3.19%, and 3.19–48.87%.

During 2015–2019, there were 31,997,726 women eligible to receive cervical cancer clinical services through the NBCCEDP, and of those eligible, 664,014 were served through the program (2.07%). When looking at the geographic distribution of the 5-year average percentage of eligible women served for cervical cancer clinical services through the NBCCEDP, there has been a greater percentage served in several counties throughout states such as Colorado, Connecticut, Kentucky, Maine, Montana, New Hampshire, New Mexico, North Dakota, South Dakota, and Washington (Figure 5).

*T*-test results indicate that there was a statistically significantly lower 5-year average percentage of eligible women served in cervical cancer hotspot clusters compared to cervical cancer coolspot clusters, 2.1% and 3.0%, respectively (tobs = 2.01, df = 357, *p* ≤ 0.05). 

**Figure 6:**
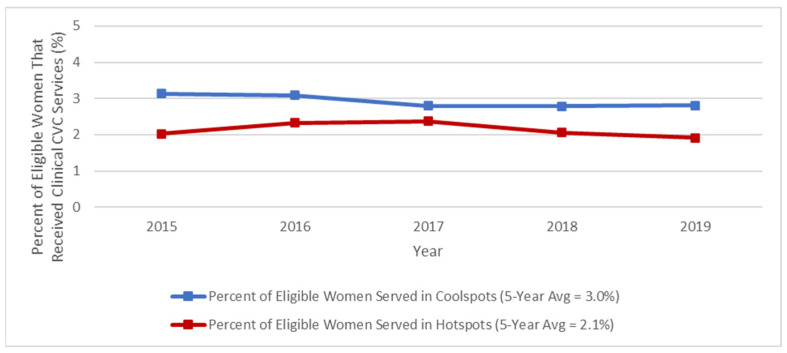
Yearly percentage of eligible women receiving clinical cervical cancer services through the NBCCEDP in cervical cancer hotspot vs. coolspot clusters, 2015–2019.

Lastly, we found that among the 235 cervical cancer coolspot clusters, the percentage of eligible women who received clinical cervical cancer services decreased from 3.1% in 2015–2016 to 2.8% in 2017–2019. Among the 124 cervical cancer hotspot clusters, the percentage of eligible women who received clinical cervical cancer services increased from 2.0% in 2015 to 2.3% in 2016–2017. However, by 2019, the percentage of eligible women who received cervical cancer clinical services in hotspot clusters had decreased to 1.9%. Overall, the annual percentages of eligible women who received clinical cervical cancer services were higher in coolspot clusters compared to hotspot clusters during each year from 2015–2019 (Figure 6).

## 4. Discussion

Formulating and using composite breast and cervical cancer burden scores that consider stage at cancer diagnosis, poverty, and screening behavior together allowed us to more accurately quantify and compare the burden of breast and cervical cancer across the United States. We found that breast and cervical cancer burden scores varied tremendously across counties, with some counties experiencing greater burden than others. In fact, some counties had breast cancer burden scores as high as +7.3, while others had cervical cancer burden scores as low as −1.6. Counties with higher burden scores are ideal for prioritizing screening intervention efforts.

Yet, in this study, we further assessed breast and cervical cancer burden scores for spatial clustering, which, to our knowledge, has never been conducted. Spatial clustering is an effective method for identifying highly burdened areas that can be used to prioritize geographic areas in greatest need of intervention [12]. Furthermore, examining spatial clusters as opposed to simple geographic distributions of cancer burden scores allows us to generate and test hypotheses regarding underlying risk and protective factors that may be common to counties within adjacent states. Ultimately, spatial clustering allowed us to pinpoint “where” there were clusters of counties with higher than average cancer burden scores (hotspots). We identified 101 breast and 124 cervical hotspot clusters across the United States, all of which were primarily in the Southern region, Idaho, and Nevada. Breast and cervical cancer hotspot clusters being predominately located in these areas is likely a reflection of these areas having higher measures across the three factors used to generate the burden scores: late stage cancer incidence, not up-to-date with screening, and a percentage with income below FPL.

While there may be multiple factors that explain the significant burden of breast and cervical cancer in the Southern region [25,26,27,28,29,30,31], we were most interested in the association between hotspot clusters and the percentage of eligible women served by the NBCCEDP. We specifically employed a *t*-test to determine if there were statistically significantly greater mean percentages of eligible women served clinical breast and cervical cancer services in clusters of counties with a higher cancer burden (hotspots) compared to a lower burden (coolspots) during 2015–2019. Contrary to our hypothesis, we found that the mean percentage of eligible women who received clinical breast and cervical cancer services was higher in coolspot clusters compared to hotspot clusters during each year from 2015–2019. We also found that the percentage of eligible women served within breast and cervical cancer hotspot clusters remained relatively stagnant or decreased during the 5-year study period. While NBCCEDP clinical services have the potential to reduce breast and cervical cancer burden, results suggest that these services are not aligned with the spatial distribution of the burden of breast and cervical cancer across counties. More specifically, we did not find that greater percentages of clinical services were delivered in counties where there was a greater cancer burden.

Current study results could inform NBCCEDP recipients’ efforts to reach and prioritize service delivery in places with the greatest breast and cervical cancer burden. NBCCEDP recipients use cancer outcome data to identify populations in greatest need, specifically those with higher late stage cancer diagnoses and mortality. Ideally, recipients then allocate resources to prioritize these populations. In alignment with these efforts, NBCCEDP recipients could choose to prioritize the distribution of clinical service delivery in identified hotspot clusters to improve programmatic impact if they have not already maximized their resources in these areas. Specifically, NBCCEDP recipients could strive to continuously increase the percentage of eligible women served in hotspot clusters. This may require increasing the number of participating screening sites, addressing system-level barriers to care, and implementing new outreach strategies to reach women eligible for screening. Implementing outreach efforts to recruit eligible women for screening through the NBCCEDP may require understanding the unique barriers of women in these hotspot clusters through recipient-led needs assessments, for which the CDC provides related technical assistance. Lastly, NBCCEDP recipients could use these hotspot clusters to educate decision-makers and help guide initiatives to increase the availability of clinical providers who deliver breast and cervical cancer screening services in these hotspot clusters.

Study results could further guide the CDC’s technical assistance and future data collection planning. For example, results indicate that it may be beneficial for the CDC to collaborate with recipients to focus on identifying strategies that are best for expanding NBCCEDP clinical services into areas with the greatest breast and cervical cancer burden. This may include the CDC identifying training to assist recipients in using available data sources for assessing cancer burden. Additionally, the CDC could consider collecting data to learn more about how often recipients are using public health data to prioritize or reprioritize the places where they are implementing the program and providing clinical services. This data could help determine how often prioritization of service delivery is needed to have the greatest impact on the breast and cervical cancer burden among program-eligible women. This data would also help to better approximate the lag time between late stage cancer incidence statistics and a targeted change in the percent of eligible women served, which would allow for an advanced spatiotemporal analysis of this relationship.

Although the study results were insightful, there were limitations. First, while eligibility for the NBCCEDP includes women who are under-insured, SAHIE data does not include these women in their estimates. Therefore, the number of NBCCEDP-eligible women used in this study was likely underestimated. Second, the age inclusions for the breast cancer screening data and the breast and cervical cancer income below poverty variables are not perfectly aligned with the NBCCEDP enrollment age criteria. Specifically, the breast cancer screening data are based on women aged 50–74, and the age inclusions for income below poverty variables are 35–64 for breast and 18–64 for cervical cancer. Yet, the NBCCEDP enrollment age criterion is 40–64 years old for breast cancer screening and 21–64 years old for cervical cancer screening. The age range for the breast cancer screening data differs from the program enrollment age criteria because it reflects the current USPSTF screening recommendations, and the age ranges used for the income below poverty data were the closest available age ranges to the program enrollment ages. This could have resulted in a slight overestimation of the contribution of the not up-to-date with breast screening and income below poverty variables toward the composite burden. Third, burden scores only account for three variables. However, cancer burden can be affected by an array of other variables, such as county-level provider density or physician shortages. Together, these limitations may result in an underestimation or overestimation of the burden of breast and cervical cancer in counties, which could have affected the location of hotspot clusters. Carrying out LISA cluster analyses using data that excludes two states (Kansas and Minnesota) also imposes limitations on study results as the clustering of counties into distinct cluster groups (i.e., hotspots and coolspots) is based on whether a county is surrounded by neighboring counties with similar rates. Thus, it is likely that the distribution of clusters across the US would be different if the counties in Kansas and Minnesota were included and assessed relative to their neighboring counties. A final limitation is that we do not consider grantee-specific factors that could affect the percentage of eligible women served, such as varying levels of funding awarded to each recipient. Therefore, differences in the percentage of eligible women served may be due to varying levels of resources available. 

## 5. Conclusions

The burden of breast and cervical cancer varies across counties in the United States. This study highlights the significant association between high burden counties and the percentage of eligible women served by the NBCCEDP. This national cancer screening program has historically served more than 16 million women across the United States and has the potential to make significant strides toward mitigating the burden of both breast and cervical cancer. However, given limited program resources, strategized targeting of highly burdened places is essential for maximizing NBCCEDP’s impact on such burdens. In efforts to improve breast and cervical cancer outcomes and ultimately reduce the burden of breast and cervical cancer, NBCCEDP recipients could: (1) use a composite approach that considers multiple factors to define and quantify breast and cervical cancer burden across counties in their states; (2) conduct routine assessments to identify clusters of highly burdened counties (hotspot clusters) relative to their state average burden score; and (3) prioritize and consistently increase clinical service delivery in the identified hotspot clusters in their states, when possible. Increasing the percentage of eligible women served in hotspot clusters aligns with NBCCEDP’s strategy to prioritize service delivery among populations disproportionately burdened by breast and cervical cancer and could also contribute toward the program’s goal of increasing the number of women served who experience higher mortality and late stage diagnoses. Ultimately, increasing the percentage of eligible women served in hotspots could help reduce the total burden of breast and/or cervical cancer in these counties, as well as reduce the geographic disparities in cancer burden seen across counties.

## Data Availability

The datasets generated and analyzed during the current study are available across multiple data sources, including two publicly available sources (Small Area Health Insurance Estimates Program (SAHIE) and the U.S. Census Bureau American Community Survey) and three restricted datasets which included county-level demographic data. The restricted datasets were subsets from larger databases including (National Breast and Cervical Cancer Early Detection Program (NBCCEDP) database; Behavioral Risk Factor Surveillance System Survey (BRFSS) database; and the United States Cancer Statistics (USCS) incidence database). The SAHIE data can be located at [https://www.census.gov/programs-surveys/sahie/data/datasets.html (accessed on 15 October 2022)]. The U.S Census Bureau American Community Survey data can be located at [https://www.census.gov/programs-surveys/acs/data.html (accessed on 3 September 2022)]. All restricted datasets can be made available from the corresponding author on reasonable request. These datasets were restricted because they represented the county-level and were unsuppressed, showing small counts.

## Appendix A

**Table A1:** Example of breast cancer burden score calculation for a single county. Cancer burden scores were generated for each US county by summing the standardized and weighted averages of late stage cancer incidence, not up-to-date with screening, and poverty measures for the 5-year period, 2015–2019. Higher burden scores reflect a greater county-level burden.

County—Level Factors	5-Year Average, 2015–2019	Mean	Standard Deviation	Standardized Value	Weight	Weighted Value
Age-adjusted late stage breast cancer incidence among women of all races aged 40–64	67.4	74.83	34.68	(67.4–74.83)/34.68 = −0.2142	45/100 = 0.45	−0.2142 × 0.45 = −0.0964
Percent of women of all races aged 50–74 not up to date with mammogram *	22.5%	29.24%	4.03	(22.5–29.24)/4.03 = −1.6724	35/100 = 0.35	−1.6724 × 0.35 = −0.5853
Percent of women of all races aged 35–64 with income below FPL in the past year	3.03%	2.95%	1.58	(3.03–2.95)/1.58 = 0.0506	20/100 = 0.20	0.0506 × 0.20 = 0.0101
						(−0.0964) + (−0.5853) + (0.0101) = −0.6716 Burden Score

* The age-adjusted BRFSS cancer screening measures are only available for 2 years of the 5-year study period (2016 and 2018), so for these measures we calculated a county-level 2-year average.
